# Systemic lupus erythematosus and cardiovascular disease

**DOI:** 10.1111/joim.13557

**Published:** 2022-08-18

**Authors:** Johan Frostegård

**Affiliations:** ^1^ Section of Immunology and Chronic Disease Karolinska Institutet Stockholm Sweden

**Keywords:** atherosclerosis, cardiovascular disease, immunity, systemic lupus erythematosus

## Abstract

The prognosis in systemic lupus erythematosus (SLE) has improved due to better treatment and care, but cardiovascular disease (CVD) still remains an important clinical problem, since the risk of CVD in SLE is much higher than among controls. Atherosclerosis is the main cause of CVD in the general population, and in SLE, increased atherosclerosis, especially the prevalence of atherosclerotic plaques, has been demonstrated. Atherosclerosis is an inflammatory condition, where immunity plays an important role. Interestingly, oxidized low‐density lipoprotein, defective clearance of dead cells, and inflammation, with a pro‐inflammatory T‐cell profile are characteristics of both atherosclerosis and SLE. In addition to atherosclerosis as an underlying cause of CVD in SLE, there are also other non–mutually exclusive mechanisms, and the most important of these are antiphospholipid antibodies (aPL) leading to the antiphospholipid antibody syndrome with both arterial and venous thrombosis. aPL can cause direct pro‐inflammatory and prothrombotic effects on endothelial and other cells and also interfere with the coagulation, for example, by inhibiting annexin A5 from its antithrombotic and protective effects. Antibodies against phosphorylcholine (anti‐PC) and other small lipid‐related epitopes, sometimes called natural antibodies, are negatively associated with CVD and atherosclerosis in SLE. Taken together, a combination of traditional risk factors such as hypertension and dyslipidemia, and nontraditional ones, especially aPL, inflammation, and low anti‐PC are implicated in the increased risk of CVD in SLE. Close monitoring of both traditional risk factors and nontraditional ones, including treatment of disease manifestations, not lest renal disease in SLE, is warranted.

## Graphical abstract

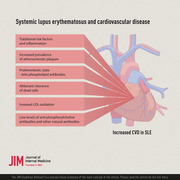

## Background and etiology

Systemic lupus erythematosus (SLE) is an autoimmune disease, where above 90% of patients are women. It is often considered a prototypic autoimmune disease, where different organ systems may be affected due to autoimmune reactions with one´s own tissue including immune complexes, autoantibodies, and cellular immunity and also inflammation in general. Symptoms show a large variation, from mild skin manifestations to life‐threatening organ failure. The disease is thus heterogenous, and diagnosis can be complicated, especially in early phases of the disease [1]. Even though the prognosis since the introduction of corticosteroids and other treatment modalities has improved considerably, there is still an increased mortality in SLE, with complications, especially lupus nephritis being important factors. Cardiovascular disease (CVD) could also be seen as another complication of the disease [1, 2].

The diagnosis of SLE is thus complex, due to the heterogeneity of the disease and its manifestations and also because the cause of the disease is poorly known. Therefore, diagnostic criteria are used. The evolution of diagnostic criteria for SLE is interesting by itself, and the most recent diagnostic criteria were from the European League Against Rheumatism/American College of Rheumatology in 2019, where positive antinuclear antibodies (ANA) were required as entry criterion and then a combination of clinical and serological/immunological manifestations and measures were the basis of diagnosis [3].

Of note, rheumatic diseases in general are criteria based, which reflects that the knowledge of their causes is relatively scarce, even though mechanisms directly causing disease are much more well defined. These criteria are likely to change in the future, and it is also likely that the borders between these diseases, especially systemic autoimmune diseases (SADs), may not be as clear as suggested by the criteria, and this is also common knowledge among clinicians. One approach is to study SADs by clustering measures including whole‐blood transcriptome and methylome, which reveals clusters among SADs, which do not strictly follow the traditional disease classifications. These clusters also tend to be relatively stable over time. However, for now, the classification criteria are useful not least for scientific studies [4].

An overactive immune system is a major feature in SLE. There are several examples of this, and there could also be different underlying disturbances, which are non–mutually exclusive.

The presence of ANA as a prerequisite for the diagnosis illustrates that nuclear material (with dead cells as the likely origin) and autoimmunity against it is a central feature of the disease [5]. Abberant and/or dysfunctional clearance of dead cells represent another important aspect of SLE [6].

An imbalance in the immune system with lower proportion of T‐regulatory cells (Tregs) is another feature of SLE and an example of the immunological aberration. Tregs are important for suppression of autoimmune immune reactions against the self. In line with this are reports demonstrating that the proportion of Tregs is lower among SLE patients as compared to controls [7, 8, 9].

In general, immunological abberations also include elevated type I interferons (IFNs). An important role of type I IFN is suggested by different lines of evidence. Gain of function variants is associated with risk of SLE and high levels are present at an early, even predisease state. Further, type I IFN is associated with higher disease activity and also nephritis and other severe manifestations of the disease [10, 11]. In addition to nuclear material from dead cells, neutrophil extracellular traps (NETs) are also induced and have been implicated in SLE, triggering IFN pathway activation [12].

Genetic and environmental but also epigenetic factors play a role in SLE, even though the pathogenesis of SLE is not well described. Among genetic factors, human leukocyte antigen (HLA) complex is the strongest, especially HLA‐DRB1 and HLA‐DR3 but hundreds of genetic loci associated with SLE have been reported. In general, these are not enough by themselves to trigger autoimmunity as in SLE, but additional environmental factors are needed. There are also relatively rare cases with a monogenetic cause of SLE [13, 14, 15, 16, 17].

Various environmental factors that may be involved in the pathogenesis of SLE include infections, especially viral, ultraviolet light, various drugs, and toxins but also psychological, psychiatric, and psychosocial factors [1]. Even gut microbiota dysbiosis has been reported in SLE [18].

These effects appear to be mediated by different epigenetic factors, such as DNA methylation, histone modification, and miRNA and long noncoding RNA expression, leading to abberant immune responses, for example, in relation to cytokines and also to breaking of tolerance against self‐antigens. Epigenetic dysregulation has been well documented in SLE, and includes DNA methylation. Especially DNA inactivation of the inactive X chromosome and hypomethylation of DNA have been reported as epigenetic hallmarks of SLE and the former is interesting given the striking female dominance in SLE [19]. A theoretically interesting and thus also clinically very relevant question is why SLE affects women so much more than men. There could be evolutionary and thus ultimate explanations behind this and also proximate ones, which directly address mechanisms involving both humoral and immunological factors.

## Epidemiology

SLE is relatively rare and the incidence of SLE shows variability, and in a meta‐analysis from 2017, from 0.3/100,000 to 23.2/100,000 person‐years were reported in different countries where the highest was in North America, while it is somewhat lower in Europe and Australia. The incidence is rising in several countries, but this may be attributable to improved diagnosis. There are also interesting differences between ethnicities, where SLE is reported to be more common in African and Arabic populations, lower among Hispanic and Asian populations, and even lower among Caucasians. The variation in estimates of prevalence appears to be even larger than incidence. The variability may be attributed to different genetic susceptibility and environmental and socioeconomic factors, but also to differences in study design, classification, and also diagnostics [20, 21, 22, 23, 24, 25].

Likewise, estimations of mortality in SLE show variation. However, a recent meta‐analysis indicates that the mortality is increased by 2.6 times. In this study, renal damage, CVD, and infection were the major factors behind this increased mortality [26]. In general, an increase in mortality by 2–3 times is suggested by different authors [20, 21, 22, 23, 24].

SLE had a much worse prognosis before treatment with immunosuppression, including cortisone, was implemented. After that, it became clear that CVD was important as a later complication of SLE and in an important study from the late 70s, a bimodal pattern of SLE was reported, where more acute complications at an early stage of disease were often followed by CVD at a later stage [27]. After that, many studies have been published where an increased risk of CVD in SLE is described and this is now established knowledge. Still, the extent and cause of this increased risk are not fully elucidated. In a previous, interesting study, it was demonstrated that among women aged 44–50 the risk increase for myocardial infarction is 50‐fold when compared to controls from the Framingham study, and further, the risk in general for coronary heart disease was increased more than sevenfold, again with historic controls after adjustment for risk factors [28].

Several other previous studies have supported the notion that the risk of CVD is increased in SLE and related to a combination of traditional and nontraditional risk factors, though there is some variation in results [25, 29–37].

An important question, given the increased risk of CVD in SLE, is which risk factors are involved. In a controlled study (with a nested case‐control design), we reported that a combination of traditional and nontraditional risk factors were implicated. Among nontraditional ones, increased levels of oxidized low‐density lipoprotein (OxLDL) was noted, and also lupus anticoagulants, which are related to antiphosholipid antibodies. Among the traditional ones, dyslipidemia was associated with CVD. However, the dyslipidemia in SLE has specific features, with low high density lipoprotein (HDL), raised triglycerides, but not raised LDL. Also, pro‐inflammatory HDL has been described [38]. Other traditional risk factors such as smoking, blood pressure, and diabetes were not associated with CVD. Both cortisone treatment and osteoporosis were associated with CVD [39].

In more recent studies, the association between CVD and SLE has been confirmed, though again, details on how strong the association is show variation [25, 40–47]. There may also have been changes over time, so that the increased risk is somewhat attenuated nowadays, due to improved prevention, treatment, and also awareness of this major SLE complication. In a recent meta‐analysis of studies conducted between 2013 and 2020, the relative risk of CVD was 2.35 and significant, which was also the case with subgroups of CVD such as myocardial infarction (MI), stroke, and peripheral artery disease [48]. The underlying cause of CVD in SLE can be related to both thrombosis, as in the antiphospholipid antibody syndrome (APS) caused by antiphospholipid antibodies (aPL), and by atherosclerotic disease, which is still not mutually exclusive. In addition, other causes include myo‐ and pericarditis and atrial fibrillation.

Of note, even though findings in different studies vary, especially in relation to traditional risk factors, these should still be in focus for prevention and treatment when possible. One example is hypertension, smoking, and diabetes, which are important risk factors for CVD, in the general population and also in CVD in SLE, though they have only recently been determined as statistically independent ones in SLE, for example in this study [25], and in a rare disease as SLE there are obvious power issues. In Table 1, independent risk factors of CVD in SLE are listed.

**Table 1:** Independent risk factors for cardiovascular disease (CVD) in systemic lupus erythematosus (SLE)

Traditional risk factors	
Age [25, 27, 45]	
Dyslipidemia (including different abberations in lipids) [25, 39, 49]	
Hypertension [25]	
Diabetes [25]	
Smoking [25]	
Nontraditional risk factors	
Antiphospholipid antibodies [25, 50–54]	
Other SLE manifestations (especially renal) [1, 2, 25, 27–37, 45]	
Cortisone treatment [25, 27, 39, 45]	
Low levels of natural antibodies including antiphosphorylcholine [55, 56, 57, 58, 59, 60, 61]	

*Note*: Commonly, in CVD in the general population, the risk factor concept is related to causation; for example, a risk marker is determined to be a risk factor when treatment of the risk marker decreases CVD (as defined by myocardial infarction (MI), stroke, peripheral artery disease, and angina). Since SLE is a rare disease, such treatment studies have not often been done, so risk markers with substantial human data are also included in the concept. There are many other promising potential risk markers for CVD in SLE, which are not included in the table, either because they are not independent of other ones, or due to a lack of substantial confirmation or being based mostly on animal experiments and not human data. References are in general from early works on the respective risk factors and/or from substantial reviews on the subject, among thousands of articles on this subject.

## aPL and CVD in SLE

In general, APS is characterized by thrombosis, both arterial and venous, and by pregnancy complications, especially miscarriage. The syndrome was first described in the late 1970s and the association with antibodies against cardiolipin was reported in 1983 by Hughes et al. [50, 51]. The role of infections, especially viral infections in aPL and APS, has been much discussed, and recently this has been highlighted in COVID‐19 infection. However, evidence that infections cause pathogenic (and not only transient) aPL is still scarce [62].

APS is divided into primary and secondary, where the first is uncommon, even though aPL per se can be determined in 1%–5% of the population, which only seldom leads to APS; a prevalence of 40–50 cases per 100,000 has been determined in a recent population‐based study [52, 53]. There is a variation in numbers as with other SADs and to some extent this is related to how the aPL are determined and classified. APS and aPL occur in SLE, the latter in about 30% of patients, with some variation in different studies [54]. Interestingly, aPL in SLE patients is more dangerous as compared to aPL in the general population and represents a major risk factor for CVD in SLE. Still, it is interesting to note that aPL is associated with myocardial infarction in young survivors, with no signs of SLE or other SADs, as determined already in studies from the 1980s [63].

Determination of aPL is also important in other SADs, not only in SLE, to decrease the risk of secondary APS [64].

aPL recognizes phospholipids, especially cardiolipin (CL), and it has become clear that phospholipids‐binding proteins play an important role, where beta‐2‐glycoprotein I is currently regarded as the most important as a cofactor for aPL, and its binding site for pathogenic aPL has been located in the N‐terminal domain. Another method is to determine lupus anticoagulants, as a measure of aPL, which interferes with phospholipids and promotes a procoagulant state. Another phospholipid that has been implicated in the antigenicity of aPL is phosphatidylserine (PS) and here, annexin A5 has been reported to be a cofactor, involved in the antigenicity [65].

There is still a discussion about the exact nature of the antigen and there are non–mutually exclusive possibilities. We reported that antibodies against oxidized but not native CL are associated with protection against atherosclerotic plaques in SLE [66]. There are also several types of autoantibodies that show similarities with aPL, such as anti‐endothelial cell antibodies and anti‐OxLDL antibodies that crossreact with anti‐CL [67], and in follow‐up studies it was demonstrated that antibodies against OxCL recognize OxLDL [68]. In a further twist to this story, OxCL has pro‐inflammatory properties that are inhibited by annexin A5 [69].

Most likely, the pathogenic effects of aPL can be divided into two, which are non–mutually exclusive: direct effects on endothelial and other cells and platelets, and interference with the coagulation system leading to a prothrombotic state.

Activation by aPL of endothelial cells has been known since the 1990s [70, 71]. Also, monocytes are activated by aPL to produce tissue factor and other compounds [72, 73]. Since platelets are also activated by aPL, this could provide a direct mechanism for thrombosis [74]. Further, thrombogenic microparticles have been reported to be induced by aPL [75]. Another aspect of this type of aPL effect is complement activation, which may promote thrombosis though mechanisms need to be elucidated more [76, 77, 78].

Effects on the coagulation system could play an important role in SLE‐related CVD through different mechanisms. Also, here are several non–mutually exclusive possibilities. One specific mechanism of how aPL can cause CVD as stroke and MI in SLE is related to annexin A5, a protein known to bind to PS, which is exposed on dying and dead cells and which functions as a danger‐associated molecular pattern (DAMP). ANXA5 is known to protect cells from damage, and forms a protective shield, binding to PS. Sera from SLE patients inhibited ANXA5 binding to endothelial cells, significantly more among SLE patients with a history of CVD than among those without. ANXA5 bound atherosclerotic plaques, especially at sites prone to rupture [79]. aPL disrupts the antithrombotic shield consisting of ANXA5, which could be one mechanism behind aPL thrombogenicity [80].

Decreased ANXA5 binding to endothelium, mediated by aPL, can be countered by IVIg. Increasing ANXA5 binding, by addition of ANXA5 itself or by use of neutralizing antibodies as in IVIg, could be a therapeutic strategy [81].

Recently, a role in thrombogenesis played by NETs and the complement system APS has also been reported [82].

Even though aPL and APS remain an important cause of CVD including thrombosis in SLE, recent studies indicate that improved treatment, with hydroxychloroquine, life‐long anticoagulant treatment, statins, and optimization of prednisolone treatment, has considerably improved prognosis and thus other causes of CVD in SLE become relatively more important [78].

## Atherosclerosis in SLE

The inflammatory nature of atherosclerosis has been known for a long time. The famous pathologists Rokitansky and Virchow described inflammation and inflammatory infiltrates, with mononuclear leukocytes, in the middle of the 19th century. It is also interesting to note that they differed in their view on the nature of this inflammation. While Rokitansky thought it was secondary, Virchow instead argued that it was a primary phenomenon. In fact, it now appears that both were right and SLE could illustrate this, since atherosclerosis and/or atherosclerotic plaques could be promoted by the disease. At the same time, atherosclerosis is a slowly developing inflammatory process in the arteries with some exceptions, most notably in the arms! It is characterized by the accumulation of dead cells, OxLDL, and infiltration of activated immune competent cells including T cells, monocytes/macrophages, and also smooth muscle cells, but not much of granulocytes, which are typically a major component of the cells in arthritis as in rheumatoid arthritis. The T cells and monocytes/macrophages show signs of activations and produce cytokines, mainly pro‐inflammatory such as interleukin 1, 6, and TNF‐alpha and accumulate close to lesions and damaged parts of the plaques [83, 84]. Calcification is also an important feature of atherosclerosis and is most likely a phenomenon related to long‐standing inflammation.

Interestingly, in the Cantos study, the inflammation hypothesis in atherosclerosis (and CVD) was supported by clinical data indicating that IL‐1 inhibition with a monoclonal antibody, canakinumab, decreased major cardiovascular events and CVD [85]. This treatment also decreased measures of inflammation including C‐reactive protein and interleukin‐6, and an IL‐6 inhibition trial is underway [86]. In line with this are results indicating that colchicine, an ancient medication that is still used in rheumatology, with anti‐inflammatory effects had a positive effect on patients with CVD including MI [87]. Still, it is interesting to note that methotrexate, which is commonly used in rheumatic diseases and also at higher doses in cancer treatment, did not influence outcome in CVD [88]. The exact type of immunomodulation and anti‐inflammatory effect are therefore essential in CVD treatment aiming at ameliorating the inflammation in plaques.

Atherosclerosis is likely not to be only a modern phenomenon, related to increasing lifespan and a sedentary lifestyle even though it is likely to be a much more pronounced phenomenon now. That atherosclerosis was present in ancient Egyptian mummies has been known for a long time [89]. Another interesting example is Ötzi, who was found in South Tyrol and died 5300 years ago, apparently shot with an arrow, which caused his death. His arteries showed signs of atherosclerosis and also calcification [90]. Though interesting, he seems to have had a genetic predisposition to atherosclerosis [91]. Still further evidence that atherosclerosis could be a feature of natural aging and immunosenescence comes from studies of hunter–gatherers, in which atherosclerosis has been detected [92].

Given the topic of this review, it is interesting to note that accumulation of dead cells in atherosclerotic plaques is a hallmark of this disease condition, which could be described as dysfunctional clearance of dead cells. Further, OxLDL is a major factor in atherosclerosis and, like dead cells, accumulates in plaques [83] and is increased in SLE [49]. Again, in addition to this, atherosclerosis, like SLE, is a chronic inflammatory condition [83, 84].

While it has been clear for a long time that the risk of CVD is increased in SLE, the underlying causes have been debated, especially if atherosclerosis, the major cause of CVD in the general population, is implicated in SLE. It is often assumed that atherosclerosis is accelerated in SLE, as in a recent review [93].

Like CVD in SLE, atherosclerosis in SLE in general is likely to be related to traditional and nontraditional risk factors, risk markers, and SLE‐specific factors. Traditional risk factors include smoking, hypertension, dyslipidemia, diabetes, but also SLE‐related factors such as disease activity and especially accumulated disease damage. Among nontraditional ones, such as an array of novel proteins and other plasma or serum factors, genetic variants, low levels of antibodies against phosphorylcholine (anti‐PC), and also circulating OxLDL have been described [55, 56, 94–102].

Still, this is a complex area, and there are different methods to determine atherosclerosis and its progress. In fact, in the only study published so far that I am aware of where SLE patients are followed during a longer time period, with matched controls, there was no increase in progress of atherosclerosis as compared to controls, as determined by ultrasound of intima‐media thickness (IMT), which is commonly used as a way to determine atherosclerosis. Of note, in the cross‐sectional study that was the basis for this prospective study, we could not determine any significant difference between IMT among age‐, sex‐, and population‐based controls as compared to SLE patients. Still, the prevalence of plaques and also of echoluscent (and potentially vulnerable) plaques was significantly increased [56]. However, in this follow‐up study, we were not able, for technical reasons, to determine the presence and/or vulnerability of plaques at follow‐up, and in general, studies that determine details of atherosclerosis measures among SLE patients as compared to controls followed for a period of time are warranted and could shed light on the nature of accelerated atherosclerosis in SLE.

## Factors in common in Atherosclerosis, CVD, and SLE: Inflammation, OxLDL, dead cells, and natural antibodies

Since the determination of T cells being present in atherosclerotic plaques [103], and also actively producing mainly pro‐inflammatory cytokines [84], their functional role in the development of CVD has been much studied and discussed. Even though much still remains to be known about the role of IL‐17 and corresponding Th17 cells and also other pro‐inflammatory T‐cell subsets, they appear to be mainly pro‐atherogenic [104, 105, 106] and human atherosclerosis including vulnerable plaques has a pro‐inflammatory T‐cell phenotype [84, 107, 108]. Tregs may suppress pro‐inflammatory effects and also have other interesting properties that could ameliorate atherosclerosis, such as inhibition of foam cell formation and induction of anti‐inflammatory macrophages, and increasing Tregs could be of interest as therapy in this context [109].

One therapeutic option in autoimmune disease, especially SLE may be to increase the proportion of Tregs, to restore the balance with effector T cells. Options include Treg‐activating compounds such as low‐dose interleukin‐2 [110, 111, 112].

The underlying cause of the low proportion of Tregs in SLE is not clear, but it may be related to the cytokine environment, promoting a decreased Treg proportion; but there are also other interesting possibilities in relation to both atherosclerosis and SLE including OxLDL and so‐called natural antibodies, especially anti‐PC but also a plasma protein, annexin A5.

LDL can be modified by different methods; both oxidation and enzymes such as phospholipases are believed to be important and non–mutually exclusive. Since oxidation is still a common denominator, the term OxLDL is commonly used. OxLDL has been in focus as a culprit in atherosclerosis since at least the late 1980s [113]. It is taken up by macrophages, which develop into inert foam cells in the atherosclerotic lesions, where they eventually die and become part of a necrotic core. OxLDL promotes cell death and has both pro‐inflammatory and immune‐stimulatory properties [83, 114, 115, 116, 117, 118]. Of note, phospholipase A2, which induces LDL oxidation and modification, is present in the arterial wall, both when atherosclerosis is established and before, where it could promote LDL modification, foam cell formation, and the ensuing immune activation [119].

Of note, OxLDL is implicated in SLE also, where it is raised as compared to controls and associated with CVD and renal disease [49]. By use of the same method, we also reported that OxLDL is raised in established hypertension [120].

An important issue is by which mechanisms and through which parts of OxLDL these effects are mediated. Inflammatory phospholipids, some with platelet activating factor (PAF)–like properties, play a role in causing pro‐inflammatory effects; lysophosphatidylcholine (LysoPC) is especially of interest [121, 122].

Another non–mutually exclusive cause of pro‐inflammatory effects and immune activation by OxLDL is phosphorylcholine (PC), which was reported in previous studies to play a role in OxLDL‐induced immune activation and production of the major pro‐inflammatory cytokine IFN‐gamma [121].

PC is the polar headgroup of phosphatidylcholine, present as a major component in cell membranes and lipoproteins [123]. During oxidation, phosphatidylcholine loses fatty acids in the sn‐2 position, which leads to bioactive properties with PC in an exposed position [124].

PC in this position promotes apoptosis, endothelial dysfunction, endoplasmatic reticulum stress, and vascular inflammation [123, 125, 126]. PC in oxidized phospholipids is atherogenic and pro‐inflammatory in mouse models [127].

An interesting example of PC as a pathogen‐associated molecular pattern (PAMP) is the polysaccharide capsule of *Streptococcus pneumoniae*. Other examples include bacteria such as Trypanoma and also nematodes, helminths, and worms [128, 129, 130, 131].

An important carrier of PC in human plasma is lipoprotein (a) (Lp[a]), which can promote arterial inflammation [132, 133]. Even though I am not aware of studies of Lp(a) in SLE, PC exposed not only on LDL (OxLDL) but also on other plasma components is raised in SLE and associated with CVD [49].

Compounds such as PC are DAMPs, which signal danger when exposed on dying and dead cells and on OxLDL. In addition, PC is also a PAMP.

Other LDL‐oxidation products and epitiopes include malondialdehyde (MDA), which is immunogenic (antibodies against MDA are abundant in humans) and which could also activate T cells both through an indirect, dendritic‐cell‐mediated and a direct effect [134].

We proposed a development of the hygiene/old friends hypothesis—low exposure to microorganisms such as nematodes, parasites, and some bacteria that have been with humans for very long time leads to low levels of anti‐PC and a lack of ability to prevent autoimmune conditions. Individuals from Kitava, New Guinea, leading a traditional life as hunter–gatherers and horticulturalists appear not to have CVD or rheumatic disease, but have strikingly high anti‐PC levels [131, 135, 136]. In line with this we have reported negative associations between anti‐PC and CVD, atherosclerosis development, and other chronic inflammatory diseases in many publications [61, 83]. These findings have been largely confirmed and also expanded upon by other researchers [60, 137–141].

Also in SLE, we reported that anti‐PC is associated with protection and in relation to prevalence of atherosclerotic plaques and other disease manifestations [55–58, 83], which has been confirmed by other researchers [59, 60]. Among subclasses and isotypes, the protective properties of anti‐PC are mainly present in IgM and IgG1 [57, 61, 142].

There are several underlying mechanisms that could explain these associations. Anti‐PC is anti‐inflammatory, inhibiting pro‐inflammatory effects on endothelial cells caused by PC‐exposing oxidized lipids [55] and promoting polarization of T cells to Tregs, both from atherosclerotic plaques and SLE patients [9]. Further to this, anti‐PC inhibits uptake of OxLDL in macrophages [143], increases clearance of dead cells [57, 144], and inhibits cell toxicity [145].

**Fig. 1:**
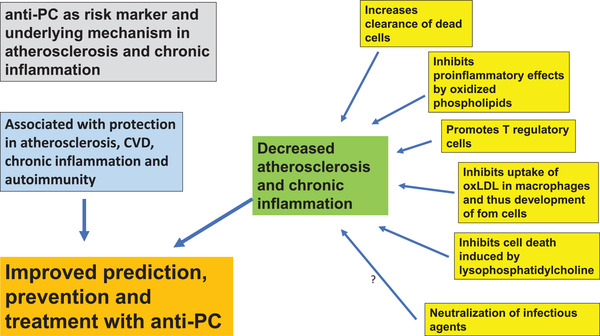
Antiphosphorylcholine (anti‐PC) as a risk marker and the underlying mechanism in atherosclerosis and chronic inflammation.

These notions are supported by mice models where raising anti‐PC is atheroprotective [146, 147]. Pneumococcal immunization leads to a significant though modest decrease in atherosclerosis and increased levels of anti‐PC among other antibodies [148]. Raising anti‐PC through immunization is also protective in both SLE [149] and rheumatoid arthritis mouse models [150]. Brown bears have much higher anti‐PC levels during hibernation, when cholesterol levels are very high and they are anuric. This is of interest since they do not show signs of atherosclerosis and also imply that anti‐PC levels can be much modulated [151]. The potential role of anti‐PC in SLE is illustrated in Fig. 1.

Also, anti‐MDA has protective properties in general and in SLE, though it is less described than anti‐PC in this context, and is also only a DAMP, not a PAMP [58, 145].

Heat shock proteins (HSPs), mainly HSP60 but also HSP90, may be of importance in atherosclerosis development, being immunogenic, and also crossreacting with bacterial HSP [152, 153]. There are HSP60‐specific T cells in atherosclerosis [154] and we demonstrated that OxLDL induces HSP60, which could indirectly activate T cells through dendritic cells (DC) [155, 156].

Another possibility, again non–mutually exclusive, is that apoB100, the protein bound to LDL, or components thereof, could be pro‐inflammatory and immunogenic and antibodies to it could be atheroprotective [157].

**Fig. 2:**
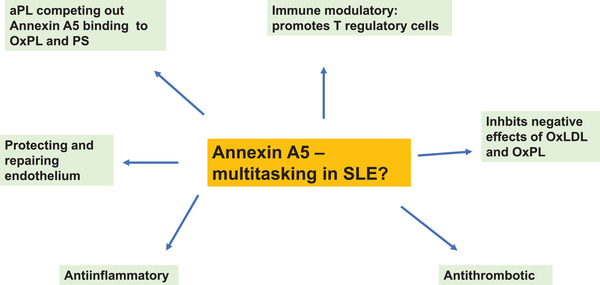
Potential role of annexin A5 in systemic lupus erythematosus (SLE).

ANXA5 also has other properties with direct relevance for SLE in addition to protecting dying cells and inhibiting effects of aPL, namely inhibition of OxLDL pro‐inflammatory properties and instead promoting development of Tregs [118]. In addition, ANXA5 has anticoagulant properties and also inhibits atherosclerosis and improves endothelial function in mouse models [158]. Further to this, ANXA5 inhibits pro‐inflammatory effects on monocytes/macrophages and endothelial cells [159]. The potential role of annexin A5 in SLE is illustrated in Fig. 2.

## Prevention and treatment

CVD is thus an important cause of both morbidity and mortality in SLE, and is caused by a combination of factors with prothrombotic and/or atherogenic properties. The traditional risk factors, such as hypertension, hyperlipidemia, and diabetes, are to be reduced through established therapies in use in general prevention of CVD. Not much data on statin treatment in SLE have been published, but recently positive data on statin use has been published, both in hyperlipidemia in SLE and APS in SLE [160]. Statins, in general, are well established as an important medication in CVD, both for primary and secondary prevention and were of course developed to decrease LDL levels by targeting the LDL receptor (LDLR).

Several different pleiotropic effects of statins have been described, which could have clinical effects in addition to lipid lowering per se [83]. These include more unspecific anti‐inflammatory effects, but also specific immunological mechanisms directly related to atherosclerosis involving defined microRNA pathways. We reported that statins repress OxLDL‐induced DC maturation, limit ensuing T‐cell activation but promote Tregs, and also repress an atherogenic HSP profile [117].

In another study, we investigated Th17 and Tregs and induction of apoptosis in cells from SLE patients as compared to controls. After activation, the proportion of Th17 versus Tregs was higher in T cells from SLE patients, an effect normalized by statins. Through machine learning, we also determined simulated interaction between statins, CRP, and IL‐6 [161].

There are even more twists to this story, relating to LDL, OxLDL, and immunity.

Proprotein convertase subtilisin kexin 9 (PCSK9) targets the LDLR, which results in increased LDL levels. PCSK9 inhibition had similar effects as statins on OxLDL‐induced plaque T‐cell activation, though microRNA pathways were somewhat different [116]. Further to this, PCSK9 is associated with disease activity in SLE and OxLDL induces PCSK9 in DC, more so among SLE patients than controls. PCSK9 could thus play an immunological role in SLE [162, 163].

These findings could have therapeutic implications in SLE, and further studies on statins and PCSK9 inhibition in SLE are warranted. However, in one randomised study, statin therapy did not influence disease in disease activity, measures of inflammation, or endothelial cell activation [164]. Still, the evidence is conflicting since statin therapy in SLE decreased inflammation markers in another study [165]. It is also possible that hydroxychloroquine treatment, which is ubiquitous in SLE, decreases the potential of additional beneficial effects in SLE.

Another important question is which role is played by corticosteroid treatment in SLE‐related CVD. There are associations between high doses of corticosteroids and CVD in SLE, but on the other hand, these patients are those with the most severe manifestations of SLE and again, it is important to optimize treatment so that SLE disease manifestations of a more acute nature are kept in check as well as possible [39, 166, 167].

Hydroxychloroquine is a cornerstone in the treatment of SLE, and interestingly, also has a role in preventing CVD. In a recent meta‐analysis, it was demonstrated that hydroxychloroquine reduces the risk of thromboembolic events by 49% [168].

There are also other treatments in SLE, including small molecule medications, which could influence CVD and atherosclerosis, though less is known about this in controlled studies. Of note, in rheumatic diseases like rheumatoid arthritis, there has been a striking development with novel biologic therapies, but in SLE this is not the case to the same extent even though belimumab and rituximab are available for the treatment and target B‐cell signalling pathways [169].

## Conclusion

The risk of CVD is high in SLE and is caused by both an increased risk of thrombosis and increased atherosclerosis, especially atherosclerotic plaques. This represents an important clinical problem but could also shed light on the inflammatory and immunological nature of atherosclerosis. A combination of traditional risk factors and nontraditional ones, including SLE‐related ones as disease activity, appear to account for this increased risk. For prevention and treatment of CVD in SLE, traditional risk factors should be targeted and SLE treatment optimized.

## Conflict of interest

J. F. is named as the inventor on patents related to annexin A5 and is the cofounder and co‐owner of Annexin Pharmaceuticals.
